# Statistics of the Lateral Operation of Lithotomy at Naples

**Published:** 1842-03

**Authors:** S. de Renzi


*Statistics of the Lateral Operation of Lithotomy at Naples.* By M. S. DE RENZI.—From the year 1821 to 1828, inclusive, 596 cases of stone were operated on, 579 of whom were males, and only 17 females. Of this number 503

recovered, and 91 died; 306 of these cases occurred in persons under 15 years of age; 231 in adults, and 59 in aged individuals. As a means of comparing the mortality from this operation, the results of the operations of lithotomy for the year 1839 are given. During this year 47 were operated on, 46 of whom were men, and 1 woman. Of these 38 recovered, and 9 died. Of this number 15 were below 15 years of age: 31 were adults, and 1 was aged.—*Ed. Med. and Surg. Journ.*, from *Il Filiatre Sebezio Giornale.*

